# Comparative Efficacy in Pertrochanteric Fractures: A Randomized Controlled Trial of the Shortest Versus Various Short-Length Cephalomedullary Nails

**DOI:** 10.1155/aort/6689145

**Published:** 2025-04-13

**Authors:** Wittawat Boonyanuwat, Nikom Noree, Pinkawas Kongmalai

**Affiliations:** ^1^Department of Orthopaedics, Faculty of Medicine, Srinakharinwirot University, Nakhon Nayok, Thailand; ^2^Department of Orthopaedics, Faculty of Medicine, Kasetsart University, Bangkok, Thailand

**Keywords:** Asian population, cephalomedullary nails, fracture fixation, orthopedic surgery, pertrochanteric fractures

## Abstract

**Background:** The optimal length of cephalomedullary nails for treating unstable pertrochanteric fractures, particularly in populations with distinct femoral anatomy, remains debated. This study compares the clinical outcomes of using the shortest effective cephalomedullary nails (170 mm) to various short length (200 mm and 240 mm), focusing on Asian patients.

**Methods:** In this randomized controlled trial, 50 patients aged 50 years or older with unstable intertrochanteric fractures (AO types 31A2-3) were randomly assigned to two groups. The shortest-nail group (*n* = 25) received a 170 mm cephalomedullary nail, while the control group (*n* = 25) received either a 200 mm or 240 mm nail based on patient height. Primary outcomes were assessed using the Harris Hip Score (HHS) at multiple postsurgery intervals. Secondary outcomes included operative time, hospital stay, hidden blood loss (HBL) (calculated using Sehat's formula), and complication rates.

**Results:** There were no significant differences in HHSs, hospital stay durations, or operative times between the two groups. The median time to fracture union was also comparable between groups (18.1 weeks vs. 18.2 weeks, *p*=0.9). However, the shortest-nail group showed significantly lower HBL (860.52 mL) compared with the control group (1183.40 mL, *p*=0.04). Complications included five cases of blade cut-out or refracture, with no significant differences between groups.

**Conclusion:** The shortest effective cephalomedullary nails (170 mm) may offer benefits, particularly in reducing HBL, compared with various short-length nails. However, this advantage may not be solely attributable to nail length, as both groups underwent similar surgical techniques and other factors, such as fracture patterns or patient anatomy, may have influenced the outcomes. While short nails are effective for treating unstable pertrochanteric fractures, larger studies with longer follow-up periods are necessary to validate these findings and assess the long-term safety and efficacy of short nails.

## 1. Introduction

Pertrochanteric fractures pose a significant challenge in orthopedic care, particularly among the elderly, where such injuries are prevalent due to factors like osteoporosis and an increased risk of falls [1–4]. Effective management of these fractures is crucial for enabling early mobilization and minimizing the risks associated with prolonged immobility, which can lead to serious complications and a decline in quality of life [5–7]. Cephalomedullary nailing has emerged as the preferred method for stabilizing pertrochanteric fractures, favored for its biomechanical stability and minimally invasive approach [8–11].

Regarding the length of the nail, long nails have been more commonly used in the past for the fixation of intertrochanteric femur fractures [12–15]. As the technique has evolved, various nail lengths have been developed, tailored to optimize patient outcomes, and reduce procedural complications. Recent advancements and studies have highlighted the effectiveness of short cephalomedullary nails in achieving outcomes comparable to those of longer nails, but with the added benefits of shorter operative times and reduced blood loss [12, 16, 17]. However, while short nails are widely used due to their advantages, some studies have reported a potentially higher incidence of secondary femoral fractures, particularly when the nail ends at the site of femoral bowing. This highlights the importance of selecting an appropriate nail length based on patient anatomy [18].

Therefore, identifying the ideal length for cephalomedullary nails remains a contentious issue, especially in the Asian population, characterized by generally shorter bone lengths compared with other regions [19–21]. Our study seeks to compare the outcomes of using the shortest effective cephalomedullary nails, designed to terminate above the site of femoral bowing, with those of various short-length nails, which might end at the femoral bowing in some patients. We hypothesize that the shortest effective cephalomedullary nail, which terminates before the femoral bowing, will provide similar functional outcomes and fracture union rates as various short-length nails without increasing complication rates.

## 2. Materials and Methods

### 2.1. Study Design and Setting

This randomized controlled trial was conducted at a tertiary referral care facility, from January 2021 to December 2022. Prior to inclusion, informed consent was obtained from all participants. The study received approval from the local ethics committee (SWUEC-251/2564F) and was conducted in accordance with the ethical standards of the 1964 Declaration of Helsinki, as revised in 2000.

### 2.2. Participants

The inclusion criteria for this study were patients aged 50 years or older requiring fixation for unstable intertrochanteric fractures (AO types 31A2-3). Exclusion criteria included patients with pathological fractures due to metastasis, impaired ambulatory function prior to the fracture, cognitive impairment, or mortality within 1 year postsurgery.

Participants were randomized into two groups on the day of surgery using a computer-generated random sequence. One group received fixation using the shortest effective cephalomedullary nail (170 mm), while the other group was treated with cephalomedullary nails either 200 or 240 mm in length, depending on the patient's height. Patients shorter than 170 cm were assigned the 200 mm nail and those taller than 170 cm received the 240 mm nail. The study utilized an intention-to-treat analysis.

### 2.3. Sample Size Calculation

The sample size was calculated to detect a clinically meaningful difference in the Harris Hip Score (HHS) between the shortest cephalomedullary nail group (170 mm) and the control group (200 or 240 mm nails). Based on prior studies examining functional outcomes after fixation of unstable intertrochanteric fractures, a mean difference of five points on the HHS was considered clinically significant. We assumed a standard deviation (SD) of eight points in both groups, based on similar orthopedic studies [22].

Using these assumptions, a power analysis was performed. To achieve 80% power with a two-sided alpha level of 0.05, a minimum of 23 patients per group was required to detect a 5-point difference in HHS between the groups. To account for potential dropouts or loss to follow-up, the sample size was increased to 25 patients per group. Thus, the total sample size for the study was 50 patients (25 in each group), which was deemed adequate to meet the study's objectives.

### 2.4. Surgical Setting and Postoperative Rehabilitation

After obtaining medical clearance, patients were promptly scheduled for surgery. In the operating room, prophylactic antibiotics were administered 30 min prior to the procedure. Under appropriate anesthesia, closed reduction was successfully achieved in all cases using a fracture table under fluoroscopic guidance, given that our study included only low-energy fractures, which typically have minimal soft tissue disruption.

The patient's skin was prepared with antiseptic in the surgical area. A skin incision, approximately 5–7 cm in length depending on the patient's size, was made proximal to the lateral aspect of the greater trochanter. The guide wire was inserted at the tip of the greater trochanter under fluoroscopic visualization. Reaming of the proximal region commenced with a 15 mm diameter.

### 2.5. Revised Sentence

Cephalomedullary nails (GAMMA II Intramedullary Nail, Changzhou Waston Medical Appliance Co., Ltd.) with a 10 mm diameter were used for all patients, with the length varying based on the assigned patient group. The insertion of the impaction blade screw, typically ranging from 85 to 95 mm, was based on the tip-apex distance, which was calculated to be less than 25 mm from fluoroscopic readings. Distal locking screws were easily inserted in a static position using an aiming guide. The entire procedure was continuously monitored with fluoroscopy.

Postoperatively, a standardized rehabilitation protocol was followed for all patients. Physical therapists assisted and encouraged bedside ambulation as part of the recovery process.

### 2.6. Primary and Secondary Outcomes

The primary objective of this study was to compare postsurgical functional outcomes using the HHS at multiple follow-up intervals (2, 6, and 12 weeks and at 6 and 12 months). HHS assessments were conducted by a single orthopedic trauma specialist using standardized clinical evaluation forms during in-person follow-up visits. The HHS, a 100-point scale evaluating hip function, was categorized as follows: < 70 (poor), 70–79 (fair), 80–89 (good), and 90–100 (excellent).

The secondary outcomes included operative time and blood loss, evaluated on the second day postsurgery. Hidden blood loss (HBL) was calculated using Sehat's formula [23]. In addition, patients underwent regular follow-ups at 2, 6, and 12 weeks and at 6 and 12 months postsurgery. Radiographs were taken during these visits to assess fracture union and monitor for complications such as implant cut-out and failure.

Normality of continuous variables was assessed using the Shapiro–Wilk test. For quantitative variables such as patient age, length of hospital stay, surgical time, fracture union time, and HHSs, data were expressed as medians and interquartile ranges (IQRs) due to non-normal distribution. Categorical variables were presented as frequencies and percentages. Comparisons between groups were conducted using the Mann–Whitney *U* test for continuous variables and the Pearson Chi-square test or Fisher's exact test for categorical variables, as appropriate.

All statistical analyses were performed using Stata 14.0 (StataCorp, College Station, TX, USA). Data collection and management were conducted using Microsoft Excel (Microsoft Corp., Redmond, WA, USA). A *p* value of < 0.05 was considered statistically significant.

## 3. Results

**Figure 1:**
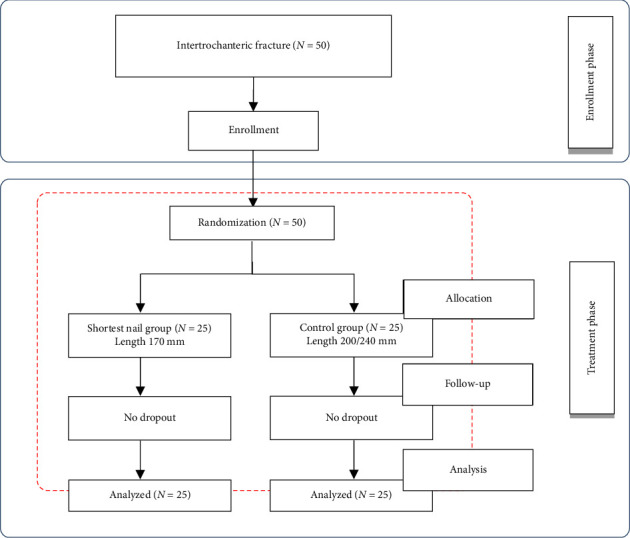
CONSORT flow diagram illustrating the progression of participants through the phases of the randomized trial.

A total of 50 patients met the inclusion criteria and were randomized into two groups. The first group, consisting of 25 patients, received fixation using the shortest effective cephalomedullary nail, measuring 170 mm (referred to as the shortest nail group). The second group, also comprising 25 patients, was treated with cephalomedullary nails either 200 mm or 240 mm in length (control group).  Figure 1 provides the flow diagram of patient randomization.

In the demographic comparison between the shortest-nail and control groups, the average ages were 63.8 years (ranging from 50 to 79) and 65.56 years (ranging from 51 to 80), respectively, showing no significant difference (*p*=0.53). The mean heights were similarly close, with the shortest-nail group at 168 cm (ranging from 160 to 180) and the control group at 169 cm (ranging from 158 to 179), also without a significant difference (*p*=0.93). The gender distribution within the groups was nearly balanced, with 10 males and 15 females in the shortest-nail group and 9 males and 16 females in the control group, resulting in nonsignificant *p* values of 0.32 and 0.48, respectively. Regarding the side of the fracture, the shortest-nail group had 12 left-sided and 13 right-sided fractures while the control group had 14 left-sided and 11 right-sided fractures, with both comparisons yielding nonsignificant differences (*p*=0.52 and *p*=0.51, respectively) (Table 1).

In comparing the shortest nail group with the control group treated with varying cephalomedullary nail sizes, the average hospital stay was 9.28 days the shortest nail group and 10.44 days in the control group, showing no significant difference (*p*=0.14) (Table 2). Similarly, the operative times for both groups were not significantly different, with the shortest nail group averaging 82 min and the control group 78 min (*p*=0.31). When evaluating functional outcomes, there was no significant difference in the HHS at any point of follow-up, including 2 weeks, 6 weeks, 12 weeks, 6 months, and 1 year postsurgery, between patients treated with the shortest effective cephalomedullary nails and those with various short-length ones. Furthermore, the time to fracture union was comparable between groups, with the shortest nail group averaging 18.1 weeks and the control group 18.2 weeks, indicating no significant difference (*p*=0.9).

**Figure 2:**
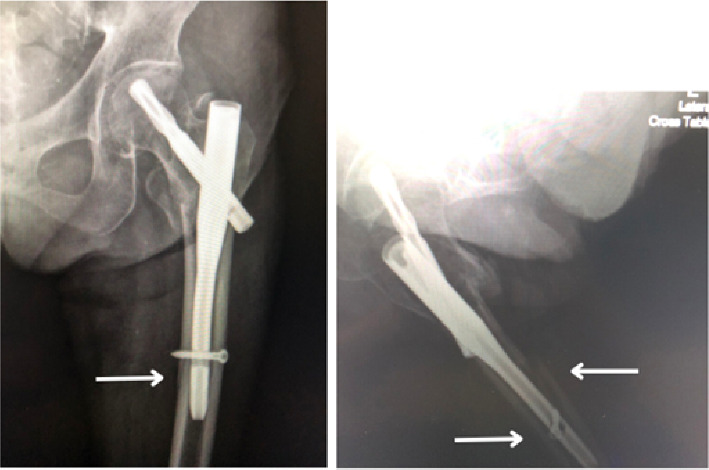
Refracture at the distal portion of the nail, marked by the arrow.

However, patients treated with the various short-length cephalomedullary nail demonstrated more HBL compared with those in the shortest effective nail group. The average HBL was 860.52 mL in the shortest nail group and 1183.40 mL in the control group (*p*=0.04). In addition, there were 5 recorded complications: two patients in the shortest nail group experienced blade cut-outs due to poor fracture reduction and high tip-apex distance, while in the control group, there was one case of refracture (Figure 2) and two cases of blade cut-out. These complications were managed with total hip replacements in four cases and refixation using a longer cephalomedullary nail in one case.

## 4. Discussion

Recent advancements in surgical techniques for pertrochanteric fractures have shown a preference for cephalomedullary nailing due to its minimally invasive approach and its biomechanical and clinical benefits. Studies have shown that short cephalomedullary nails are comparable to longer ones in terms of axial and torsional stiffness and ultimate axial strength [24]. Clinically, research supports the efficacy of short nails in managing pertrochanteric fractures without significant differences in postoperative clinical outcomes [25, 26]. In addition, short nails may offer advantages such as shorter operation times [17], reduced perioperative blood loss [12, 16], and lower rates of postoperative thigh pain [18]. However, their potential association with periprosthetic fractures depends on anatomical factors, particularly whether the nail length appropriately accounts for femoral bowing to avoid creating a stress riser. A meta-analysis by Cinque et al. [27] highlighted that short nails are associated with significantly less blood loss and shorter operative times than long nails, without significant differences in transfusion rates, implant failures, reoperation rates, or periimplant fractures, supporting their use in most intertrochanteric femur fractures.

Despite debates on the optimal length of cephalomedullary nails, the search for the shortest effective length continues, aiming to balance stability and minimize complications. No level 1 randomized controlled trials have yet defined the minimum effective length for these treatments. Our study is the first to show no significant differences in HHSs, hospital stay lengths, operative times, or fracture union rates among groups treated with cephalomedullary nails of the shortest effective length (170 mm) and various short-length variants (200 and 240 mm). Although our results suggest a potential reduction in HBL with shorter nails, this difference should be interpreted with caution. Given that the surgical techniques were largely identical between the two groups—both procedures involved the absence of reaming and used the same distal locking technique—the observed variation in blood loss is unlikely to be solely due to nail length. It is more likely that this difference may have been influenced by other factors, such as variations in fracture patterns or individual patient anatomy.

**Figure 3:**
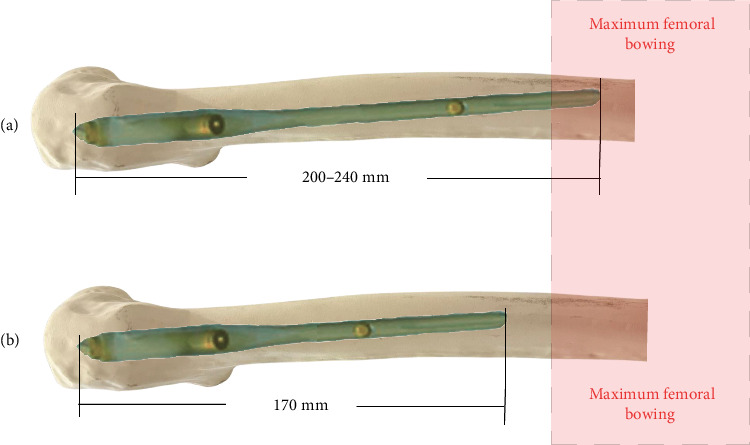
(a) The shortest nail group with the nail ending before the maximum femoral bow. (b) A nail ending at the femoral bow, which could create a stress riser and increase the risk of subsequent fractures.

Literature, including the research by Li et al. [15], indicates that shorter nails may be associated with a higher risk of secondary periprosthetic femoral fractures, possibly due to a mismatch with the femoral bow, leading to anterior cortical penetration. This is especially pertinent in Asian populations, which tend to have more pronounced anterior femoral bowing and shorter femoral bones [28, 29]. Theoretically, a nail ending at the femoral bow could create a stress riser, increasing the risk of subsequent fractures [30, 31] (Figure 3) Thus, shorter nails are designed to avoid extending beyond the apex of the proximal femur's anterior bow. Our findings endorse the shortest effective nail as a feasible option, potentially reducing operative time and HBL. However, nail length selection should be personalized, considering each patient's anatomical and clinical characteristics.

Although previous studies have reported a 1-year mortality rate of approximately 15%–30% in patients with hip fractures [32–34], we did not observe any deaths during our study period. This could be attributed to the relatively small sample size, exclusion of patients with significant comorbidities or impaired ambulatory function prior to the fracture, and the comprehensive perioperative care provided at our tertiary center. Notably, a recent study [35] has also reported a declining trend in hip fracture mortality rates due to advancements in surgical techniques, perioperative management, and multidisciplinary rehabilitation programs, which align with our findings.

Our study has several limitations, including a relatively small sample size and its execution within a single center, which may restrict the generalizability of our findings to broader populations. Although we conducted follow-ups with patients 12 months postoperation, this period may not suffice to fully evaluate long-term outcomes or detect late-onset complications, essential for a comprehensive understanding of the treatment's enduring effectiveness. The absence of blinding among participants and surgeons might lead to bias in evaluating outcomes. Moreover, future research should consider factors such as patient height, bone density, and specific fracture configurations. Addressing these elements in future studies could yield deeper insights into the long-term efficacy and safety of using short cephalomedullary nails in the treatment of pertrochanteric fractures.

## 5. Conclusion

Our randomized controlled trial demonstrates that cephalomedullary nails of the shortest effective length (170 mm) are effective in treating unstable pertrochanteric fractures, providing clinical outcomes comparable to various short-length nails (200 and 240 mm). Although our findings suggest a potential reduction in HBL with shorter nails, these results should be interpreted cautiously, as the similarity in surgical techniques between groups and other patient-specific factors may have influenced the outcomes. Moreover, the choice of nail length should be tailored to each patient's anatomical characteristics, considering factors such as femoral bowing and bone geometry to minimize the risk of complications like periprosthetic fractures.

## Figures and Tables

**Table 1:** Demographic characteristics of participants by group.

	Shortest-nail group	Control group	*p* value
Age (years)	63.8 (50–79)	65.56 (51–80)	0.53
Height (cm)	168 (160–180)	169 (158–179)	0.93

*Sex*			
Male	10	9	0.32
Female	15	16	0.48

*Side of fracture*			
Left	12	14	0.52
Right	13	11	0.51

**Table 2:** Comparative outcomes between the shortest nail group and control group.

	Shortest-nail group	Control group	*p* value
Hidden blood loss (mL)	860.52 (252–2368)	1183.40 (288–2863)	0.04
Operative time (mins)	83 (50–115)	78 (55–107)	0.31
Hospital stay (days)	9.28 (5–14)	10.44 (5–14)	0.14
Time to union (weeks)	18.1 (12–24)	18.2 (12–23)	0.9
HHS at 2 weeks	48.5 (40–60)	51 (41–60)	0.13
HHS at 6 weeks	49.6 (41–59)	49.5 (40–59)	0.96
HHS at 12 weeks	65.3 (52–80)	65.12 (50–78)	0.22
HHS at 6 months	80.4 (70–90)	81.8 (70–90)	0.68
HHS at 1 year	80 (70–95)	82 (70–95)	0.53

## Data Availability

The data that support the findings of this study are available on request from the corresponding author. The data are not publicly available due to privacy or ethical restrictions.
